# Antimicrobial Resistance Pattern and Empirical Antibiotic Treatments in Neonatal Sepsis: A Retrospective, Single-Center, 12-Year Study

**DOI:** 10.3390/antibiotics12101488

**Published:** 2023-09-27

**Authors:** Chiara Minotti, Antonella Di Caprio, Laura Facchini, Luca Bedetti, Francesca Miselli, Cecilia Rossi, Elisa Della Casa Muttini, Licia Lugli, Laura Luppi, Filippo Ferrari, Alberto Berardi

**Affiliations:** 1 Neonatal Intensive Care Unit, University Hospital of Modena, 41124 Modena, Italy or chiara.minotti@unibas.ch (C.M.); laurafacchini94@gmail.com (L.F.); luca.bedetti87@gmail.com (L.B.); miselli.fnc@gmail.com (F.M.); rossi.cecilia@aou.mo.it (C.R.); dellacasa.elisa@aou.mo.it (E.D.C.M.); lugli.licia@aou.mo.it (L.L.); alberto.berardi@unimore.it (A.B.); 2 PhD Program in Clinical Research, University Children’s Hospital Basel, University of Basel, 4031 Basel, Switzerland; 3 PhD Program in Clinical and Experimental Medicine, University of Modena and Reggio Emilia, 41124 Modena, Italy; 4 Clinical Microbiology Unit, University Hospital of Modena, 41124 Modena, Italy

**Keywords:** antimicrobial stewardship, neonatal infection, neonatal sepsis, infection prevention and control, antimicrobial resistance

## Abstract

Neonatal sepsis is an important cause of morbidity and mortality in neonatal intensive care units (NICUs). Continuous evaluation of antimicrobial resistance (AMR) profiles is advised to implement antimicrobial stewardship (AMS) programs and establish effective empiric antibiotic protocols. AMS may reduce AMR in NICUs and improve sepsis outcomes. In this retrospective observational study, we report data on culture-positive neonatal sepsis, assessing differences after the implementation of an AMS program (2011–2016 vs. 2017–2022). A total of 215 positive bacterial cultures from 169 infants were retrieved, with 79 early-onset (36.7%) and 136 late-onset (63.3%) sepsis episodes. Frequent causative agents for early-onset sepsis were *S. agalactiae* and *E. coli*, all susceptible to empiric treatment. Late-onset sepsis was mainly caused by *Enterobacterales* and *S. aureus*. Aminoglycosides, cefotaxime, and piperacillin-tazobactam resistance among *Enterobacterales* was substantially low; *S. aureus* was mostly susceptible to oxacillin and vancomycin. There were no differences in mortality and multidrug-resistant pathogens rates between the two study periods. There were five episodes of fungal late-onset sepsis, mostly due to *C. albicans*, of which one was fatal. The microbial distribution pattern and AMR profiles overlapped with other European studies. Because susceptibility patterns are rapidly changing worldwide, with the emerging threat of Methicillin-resistant *S. aureus* and extended-spectrum beta-lactamases producers, infection prevention and control practices and AMS strategies require continuous optimization to limit selection pressure and AMR escalation.

## 1. Introduction

Neonatal sepsis is an important cause of morbidity and mortality among infants in neonatal intensive care units (NICUs), in particular those born preterm or with very low birth weight (VLBW, birth weight < 1500 g). Indeed, the survival of VLBW infants has increased following the advances in critical care, but prolonged hospital stays and the need for invasive procedures (such as mechanical ventilation or use of central venous lines) are associated with increased risks of bloodstream infection [1,2,3,4,5]. Furthermore, pathogens causing neonatal infections and their antimicrobial susceptibility patterns may change over time and across different countries [1].

Italy and Greece are the two European countries with the highest number of death and long-term disabilities related to MDR resistance rates, particularly in the age group under one year [6]. Therefore, surveillance of antimicrobial resistance (AMR) within intensive care units plays a primary role in these countries.

International guidelines recommend administering beta-lactams (usually ampicillin or penicillin) plus an aminoglycoside as empirical therapy of early-onset sepsis (EOS) [7,8,9]. This association provides coverage against *S. agalactiae* and *E. coli*. The first-line treatment of late-onset sepsis (LOS) should include a beta-lactam (i.e., a penicillinase-resistant penicillin such as oxacillin or nafcillin) plus an aminoglycoside. A third-generation cephalosporin should be added in suspicion of meningitis because of increased central nervous system (CNS) penetration and higher activity against Gram-negative pathogens. Second-line treatments include glycopeptides in contexts where Gram-positive pathogens (such as methicillin-resistant *S. aureus*, MRSA, and coagulase-negative *Staphylococci*, CoNS, responsible for 48–55% of sepsis in VLBW) are predominant [2].

Although antibiotic therapy plays a key role in the treatment of neonatal infections, some potential adverse effects should be considered, especially for uninfected premature neonates [10,11]. Prolonged antibiotic use (over 5 days) increases the risk of necrotizing enterocolitis, bronchopulmonary dysplasia, and invasive fungal infections [12]. Moreover, unnecessary antibiotics increase the selection pressure on bacteria and result in the rapid increase worldwide of multidrug-resistant organisms (MDROs) such as gentamicin-resistant Gram-negative bacteria, MRSA, extended-spectrum beta-lactamase producers (ESBL), carbapenem-resistant *Enterobacterales* (CRE), and vancomycin-resistant *Enterococci* (VRE) [13]. Sepsis caused by MDRO increases the risk of morbidity and mortality, largely because empiric antibiotic therapies may not be effective. The growing spread of AMR represents a major threat, with the risk of MDRO sepsis having increased logarithmically over the past two decades [3,14]. Risk factors for MDRO colonization and infection include, again, prolonged length of stay, indwelling medical devices, and prolonged antibiotic exposure [3]. Therefore, the assessment of antimicrobial susceptibility patterns and neonatal outcomes over time is important to guide the selection of appropriate antibiotic therapies. Continuous assessment of the predominant pathogens and their AMR profiles should be conducted; in addition, effective empiric antibiotic protocols, consistent with antimicrobial stewardship (AMS) programs, should be established [12]. Indeed, the implementation of an AMS strategy may reduce AMR in the NICU, potentially improving sepsis outcomes.

In this study, we aimed to report data on the local epidemiology of pathogens causing bacterial and fungal neonatal sepsis and their antimicrobial susceptibility over 12 years. A secondary aim was to assess any eventual changes in resistance patterns and mortality before and after the implementation of an AMS program.

## 2. Results

### 2.1. Bacterial Sepsis Episodes

During the study period, 215 bacterial sepsis episodes occurred (EOS, *n* = 79, 36.7%; LOS, *n* = 136, 63.3%) among 169 infants (period 1: 91 episodes among 78 neonates; period 2: 124 episodes among 91 neonates, Table 1). More than half of the episodes (121/215, 56.2%) occurred in 95 VLBW infants and 130/215 episodes (60.4%) occurred in 101 infants born under 34 weeks of gestation. One hundred and ten episodes occurred among females (51.1%). Among 215 isolates, 184 (85.6%) grew from blood culture only, 18 (8.4%) from CSF only, and 13 (6.0%) from both blood and CSF. Overall sepsis-associated mortality was 13% (22/169 infants).

#### 2.1.1. Period 1 (from 2011 to 2016)

Overall, during period 1 there were 91 episodes of sepsis (EOS, *n* = 22, 24.2%; LOS, *n* = 69, 75.8%) in 78 infants (Table 1). Sepsis-associated mortality occurred in 16.7% of infants (13/78). Most episodes occurred among neonates born under 34 weeks of gestation (55/91, 60.4%) or VLBW infants (53/91, 58.2%). Gram-positive pathogens were yielded in 48 episodes (52.7%), whereas Gram-negative pathogens were yielded in the remaining 43 episodes (47.3%, Table 1).

##### EOS and LOS

Most EOS episodes (14/22, 62.6%) were due to Gram-positive bacteria. The most frequently isolated pathogen was *S. agalactiae* (*n* = 9, 64.2%), followed by *E. faecalis* (*n* = 2), *L. monocytogenes* (*n* = 2), and *S. aureus* (*n* = 1). Eight EOS episodes (36.4%) were caused by Gram-negative pathogens: among these, *E. coli* was the most common (*n* = 6 cases). *K. pneumoniae* accounted for the remaining two cases. The resistance patterns of single pathogens are reported in Table 2.

More than half of LOS episodes (35/69, 50.7%) were due to Gram-negative pathogens, namely *Enterobacterales* (mostly *K. pneumoniae*, *n* = 13, and *E. coli*, *n* = 10). Thirty-four (49.3%) of 69 LOS episodes were due to Gram-positive bacteria (mostly *S. aureus*, *n* = 13 and *E. faecalis*, *n* = 12). Table 2 and Table 3 offer details on resistance patterns of Gram-positive and Gram-negative isolates in EOS and LOS during periods 1 and 2. 

#### 2.1.2. Period 2 (from 2017 to 2022)

Overall, during period 2 there were 124 episodes of sepsis (EOS, *n* = 17, 13.7%; LOS, *n* = 107, 86.3%) in 91 infants (Table 1). Sepsis-associated mortality occurred in 9.9% of infants (9/91). Most episodes occurred among neonates born under 34 weeks gestation (75/124, 60.5%) or VLBW infants (68/124, 54.8%). Gram-negative pathogens were yielded in most cases (67 episodes, 54.0%), whereas Gram-positive pathogens were yielded in the remaining 57 episodes (46.0%, Table 1).

##### EOS and LOS

Eleven out of 17 EOS episodes (64.7%) were due to Gram-positive bacteria (mostly *S. agalactiae*, *n* = 8). Six episodes (35.3%) were caused by Gram-negative pathogens (*E. coli*, *n* = 4, *P. aeruginosa*, *n* = 2, Table 2).

Among the 107 episodes of LOS, 61 (57.0%) were due to Gram-negative pathogens (mostly *K. pneumoniae*, *n* = 21, *E. coli*, *n* = 13, *E. cloacae n* = 10, and *P. aeruginosa n* = 8); the remaining 46 episodes (43.0%) were caused by Gram-positive pathogens (mostly *E. faecalis*, *n* = 19, and *S. aureus n* = 19, Table 3).

### 2.2. Fungal Sepsis Episodes

There were five episodes of fungal LOS overall, occurring in VLBW infants born under 34 weeks of gestation.

During period 1, there were three cases of LOS by multisensitive *C. albicans* (60%), Sepsis-related mortality occurred in one patient (33.3%).

During period 2, there were two cases of fungal LOS overall (40%): one case by *C. parapsilosis* and one by *C. glabrata*, both mulitisensitive. Both infants survived.

### 2.3. Pathogen Susceptibility Patterns and Outcome Comparison between Study Periods 1 and 2

Considering the causative pathogens of EOS, Gram-positive bacteria were all susceptible to the empiric treatment over the two study periods. Among Gram-negatives, only one ESBL-producing *E. coli* was yielded in period 1; moreover, there was a low and comparable proportion of resistance to gentamicin, cefotaxime, and piperacillin-tazobactam among *Enterobacterales* over the two study periods.

As for LOS episodes, there were six episodes due to MRSA (period 1, *n* = 2, period 2, *n* = 4) and no cases of VRSA or VRE, CRE, CRPA or CRAB. Moreover, there were only a few strains of ESBL-producing *K. pneumoniae* (period 1, *n* = 1, period 2, *n* = 2), *E. coli* (period 1, *n* = 2, period 2, *n* = 1), and *K. oxytoca* (period 2, *n* = 1). Despite variations, resistance to aminoglycosides, cefotaxime, and piperacillin-tazobactam among *Enterobacterales* was substantially low. Overall, there were no differences in the rates of MRSA (2/91, 2.2% versus 4/124, 3.2%, *p* = 0.6) and ESBL strains (4/91, 4.3% versus 4/124, 3.2%, *p* = 0.6) across the two study periods. (Table 4).

As summarized in Table 4, sepsis-associated mortality did not differ significantly between the two study periods (13/78 infants, 16.7% versus 9/91 infants, 9.9%, *p* = 0.2).

For the secondary outcomes, there was a substantial overlap between periods 1 and 2 concerning the rate of sepsis episodes occurring in infants born under 34 weeks gestation (55/91, 60.4% versus 75/124, 60.5%, *p* = 0.9) and in VLBW infants (53/91, 58.2% versus 68/124, 54.8% *p* = 0.6).

## 3. Discussion

The present study, conducted in a single Italian center from 2011 to 2022, reported that the main causative agents of EOS were Group B *Streptococcus* and *E. coli*, while the most frequently responsible pathogens for LOS were *K. pneumoniae*, *E. coli*, *E. faecalis* and *S. aureus*. Microbiological findings did not vary across the two study periods and are in line with previous Italian and European data [1,2,15,16,17,18,19,20,21,22,23]. Following the most recent evidence on AMR, the greatest burden of disease is determined by third-generation cephalosporin-resistant *Escherichia coli*, MRSA and third-generation cephalosporin-resistant *K. pneumoniae*. Infants under one year and the elderly had the greatest overall age-group-specific burden [21]. According to the 2016–2020 report on the health burden of infections with antibiotic-resistant bacteria in Europe of the European Centre for Disease Prevention and Control (ECDC), adjusted for population size, the overall burden of AMR infections was highest in Greece, Italy and Romania [22]. According to the Surveillance Atlas of Infectious diseases of ECDC for healthcare-associated bloodstream infection in ICUs, in Italy there was the highest incidence density in 2019, with 4.8 infections/1000 patients/day. However, the overall burden was estimated to fall in 2020.

As far as concerns about NICUs, on a global scale, outbreaks are increasingly caused by ESBL-*K. pneumoniae*, MRSA and VRE [23]. Consistent with the current literature, in our study sepsis episodes mostly occurred in high-risk infants, such as those born under 34 weeks of gestational age and VLBW infants, and rates unchanged over time. Sepsis-related mortality rates were also comparable over the two study periods.

Similar to some European or Italian studies [2,15], *S. agalactiae* and *E. coli* were the most common pathogens in EOS and were usually susceptible to the empiric antibiotic treatment (ampicillin plus gentamicin) recommended in our center as first line. On the other hand, the main Gram-positive pathogens responsible for LOS were generally susceptible to penicillinase-resistant penicillin (i.e., oxacillin for *S. aureus*) or glycopeptides (for *S. aureus* and *E. faecalis)*. Despite variations, resistances to aminoglycosides, cefotaxime, and piperacillin-tazobactam among *Enterobacterales* were substantially low, whereas pathogens causing LOS were mostly susceptible to our first line antibiotic regimens. Our findings are similar to those reported in a recent study conducted in Northern Europe in which vancomycin and cefotaxime (96.1%), vancomycin and gentamicin (97.0%), and cloxacillin and gentamicin (38.1%) were used as first line antibiotics in LOS [15].

In our study, the rate of neonatal sepsis episodes caused by MDR pathogens was low overall and well below 5%, without differences between the two study periods. Such results are not as alarming as those recently described by Fang and colleagues for MDR prevalence in EOS and LOS in a Tertiary Care Children’s Hospital in China [24]. This recent study reported high rates of MDR pathogens causing EOS and LOS (almost 60%), with MRSA reaching 33.3% of cases of LOS and carbapenem-resistant *K. pneumoniae* reaching almost 70% of cases. Such results reveal an unprecedented, growing prevalence of MDR strains responsible for neonatal sepsis, thus advocating for the need for effective prevention measures and treatment options, already envisaging colistin for MDR Gram-negative bacteria, and glycopeptides for staphylococcal infections. Moreover, another recent study by Zhu et al. reported a high proportion of ESBL *E. coli* LOS in preterm infants, with a mortality rate of twice as compared to sepsis caused by non-ESBL strains [25].

Wide spectrum, prolonged antibiotics lead to the spread of MDR pathogens in the NICU, with associated risks of adverse outcomes (i.e., invasive candidiasis) [12]. A 2021 Cochrane systematic review evaluated the existing literature concerning different antibiotic regimens for LOS. Investigators included five randomized controlled trials comparing different regimens and concluded that the superiority of one regimen over another (in terms of beneficial and harmful effects) could not be demonstrated [26]. Moreover, another recent systematic review explored the efficacy of empiric antibiotic treatment of LOS due to *Enterobacterales*. Less than 50% of *Klebsiella* spp., *E. coli*, and *Enterobacter* spp. were susceptible to the standard empiric treatments recommended by WHO (ampicillin plus gentamicin as first-line treatment and third-generation cephalosporin as second-line treatment). Thus, a revised guideline for empiric antibiotic treatment of neonatal sepsis was advocated [27].

Considering fungal neonatal sepsis, *Candida* spp. are one of the most important pathogens in infants admitted to NICUs, determining invasive infection with a high burden of morbidity (including late neurodevelopmental sequelae) and mortality, representing the third cause of LOS in NICUs [28]. *Candida* spp. should be regarded as true pathogens even when identified from only a set of blood cultures because of its relatively low sensitivity [29]. Cases retrieved during the study period were few and only one of them was fatal. They were mostly due to *C. albicans* (60%), which is still the prevalent causative pathogen among fungi, even though a reduction has been reported over the last decade, with *non-albicans* species being now prevailing [30].

Overall, in the current 12-year study period MDR pathogens causing neonatal sepsis were infrequent. We were therefore unable to demonstrate a decrease in resistance rates from period 1 to period 2, although an AMS program had been implemented over time. AMS and IPC measures are paramount to prevent or limit the spread of MDR pathogens; as already demonstrated for adult patients, a high colonization pressure is a risk factor for MDR infections acquired in NICU. Furthermore, new, non-culture methods for resistome determination (i.e., molecular multiplex PCR for detection of AMR) have the ability to detect causative agents directly in the sample, and faster than the antimicrobial susceptibility testing systems used in this study. Assessing the genetic resistance of infective agents is an opportunity for future, rapid, accurate diagnosis, that can be achieved in a few hours [31]. This technique will possibly represent an important contribution to promptly obtaining laboratory results and resistance information, as well as to monitoring infections.

### Limitations

This study has several potential limitations. First, the study design is retrospective, and encompasses a relatively short period at a single center. Second, the length of antibiotic treatment and hospital stay were not included among the outcomes. Third, we did not measure the compliance to the IPC practices in place, as this was beyond the aims of the study. Last, we excluded CoNS bacteremia (whose diagnosis is sometimes difficult) to focus on pathogens that are more commonly responsible for severe infections. Because our center is a level-three care facility, the population differs from hospitals offering different levels of care. Therefore, our results may not be generalizable to the neonatal population in terms of patient characteristics, NICU environment, and treatment practices. Nevertheless, our data allow benchmarking with centers having higher (such as some Asian countries) or comparable (such as some European studies) prevalence of MDR infections.

## 4. Materials and Methods

### 4.1. Study Design, Population and Setting

This is a single-center, retrospective, observational, pre-post, quasi-experimental study, conducted at the intermediate care unit and the NICU of Policlinic University Hospital of Modena (Italy). This is a high-volume level-three facility, with inborn neonates accounting for most admissions. The NICU contains 20 cots, receives approximately 450 admissions per year, and the medical staff consists of 12 physicians. The population of infants on the ward mainly includes premature newborns, with special attention to post-surgery patients, coming from other wards or infants being transferred from other facilities, with surveillance cutaneous and mucosal swabs being taken on admission for inborn and outborn patients alike, and the possibility of cohorting.

In our center, strategies to reduce unnecessary antibiotics have been implemented for years. Since 2009, late preterm or full-term neonates at risk of EOS have been managed through a serial physical examination approach, which is associated with low rates of unnecessary antibiotic exposure [32]. Our center has a major, coordinating role in the regular surveillance of all sepsis episodes occurring in Emilia-Romagna, a Northern Italian region [33,34] with approximately 35.000 live births/year. Furthermore, an AMS program for EOS and LOS was first introduced at the end of 2014. Since 2016, this has been implemented in the NICU [35] and then shared with the entire region, following repeated audits and meetings, with discussion of the available literature [34].

Before the implementation of AMS, empirical treatment of neonatal sepsis encompassed ampicillin plus an aminoglycoside (for EOS) and teicoplanin plus an aminoglycoside (for LOS). Third-generation cephalosporins or carbapenems were administered according to the physician’s judgment—if the blood cultures were sterile, and antibiotics were never discontinued early and continued for 7 or more days. The duration of the antibiotic treatment was decided by the attending physician on a case-to-case basis.

Since AMS, empiric antibiotics are discontinued early (within 36–48 h) if the blood culture is sterile; if blood culture yields pathogens, broad-spectrum antibiotics are shifted to narrow-spectrum regimens, that are selected according to the pathogen antimicrobial susceptibility. According to disease severity, targeted antibiotic courses last from 5–10 days (Gram-positive sepsis) to 14/21 days (Gram-negative sepsis/meningitis). Moreover, the use of third-generation cephalosporins is restricted to suspected meningitis whereas carbapenems are used to treat MDR pathogens.

Currently, the recommended first-line empirical antibiotics at our facility are as follows:

1. Ampicillin + gentamicin for EOS;
2. Oxacillin + aminoglycoside (usually gentamicin or amikacin, for LOS);
3. LOS in infants colonized by MRSA: vancomycin + aminoglycoside;
4. LOS in infants colonized by an ESBL: oxacillin + meropenem;
5. Serious abdominal infection/perforation: oxacillin + aminoglycoside + metronidazole (alternatives: piperacillin-tazobactam or meropenem) [9,36].

Intravenous caspofungin or lyposomal amphotericin B represent the empirical treatments of choice for fungal infection, to be shifted to fluconazole in case of *C. albicans* isolation. Since 2008, fluconazole at a dose of 3 mg/kg/day EOD is administered as a prophylaxis of neonates under 1000 g for the first six weeks of life.

Training AMS sessions have been offered to attending physicians, nurses and residents. Hand-washing and other infection prevention and control (IPC) practices (such as bundles for central catheter insertion and care, and Kangaroo care) were already in place.

### 4.2. Inclusion and Exclusion Criteria

The microbiology laboratory database was searched for bacterial isolates grown in blood and/or cerebrospinal fluid (CSF) cultures from live-born infants of all gestational age neonates relative to the 12-year study period, from 1 January 2011 to 31 December 2022. Only cases of sepsis, in which blood or CSF culture positivity was associated with antibiotic treatment of any duration, were included. Cases of sepsis by *Candida* spp. were considered separately. All potential contaminants (CoNS, *Micrococci*, *Aerococcus* species, *Corynebacterium* species, *Propionibacterium* species, *Bifidobacterium* species, and *Bacillus* species), grown in a single culture, were excluded from the analysis for the difficulty in discriminating true infection from contamination and to focus on true bacterial sepsis episodes. When infants had two or more positive cultures within 5 days, also from different sites (i.e., blood and CSF), only the first culture was included for analysis. For each positive isolate identified in the laboratory database, study staff collected strictly anonymous neonatal demographics, data on antibiotic treatment, and outcomes (mortality) from patients’ electronic medical records.

The impact of the intervention was assessed by comparing 6 years before and after the AMS implementation.

### 4.3. Definitions

Early-onset (EOS) and late-onset sepsis (LOS) were defined as a positive blood or CSF culture in an infant aged less than 72 or ≥72 h of life, respectively. Sepsis-associated mortality was defined as death occurring within 7 days from its onset [37].

1. MRSA: Methicillin-resistant *S aureus*;
2. VRSA: Vancomycin-resistant *S. aureus;*
3. VRE: Vancomycin-resistant *Enterococci;*
4. ESBL-producing *Enterobacterales*: extended-spectrum beta-lactamases producers;
5. CRE: Carbapenem-resistant *Enterobacterales*; *Enterobacterales* that test resistant to imipenem and/or meropenem or produce a carbapenemase;
6. CRPA: Carbapenem-resistant *Pseudomonas aeruginosa*;
7. CRAB: Carbapenem-resistant *Acinetobacter baumannii* [38].

### 4.4. Outcomes

The primary outcomes were:

1. Differences in bacterial sepsis-associated mortality between the two study periods.
2. Changes in pathogens susceptibility patterns to the most used antibiotics after the implementation of AMS. Thus, we described the rate of isolation of MRSA, VRSA, VRE, ESBL, CRE, CRPA and CRAB across the two study periods, respectively.

The secondary outcomes were as follows:

1. Differences between the two study periods in the number of infection episodes in patients under 34 weeks gestation;
2. Differences between the two study periods in the number of infection episodes in VLBW infants.

### 4.5. Antimicrobial Susceptibility Testing

Identification of the species level and antimicrobial susceptibility testing of the isolates was performed by the VITEK MS and VITEK 2 automated system (BioMérieux, Grassina, Italia, S.p.A.), respectively, according to the interpretive criteria of the European Committee on Antimicrobial Susceptibility Testing.

### 4.6. Statistical Analyses

Categorical variables were reported as a number and frequency (%). Pearson’s chi-squared tests were used for the comparison of categorical variables between the two study periods. IBM^®^ Corp SPSS Statistics, Armonk, NY (USA). Version 26.0 provided the results of the statistical analysis; a *p*-value ≤ 0.05 was considered statistically significant.

### 4.7. Ethical Considerations

Data collected were fully anonymous and did not include any identifiable information of patients or caregivers. The local Institutional Research Ethics Board (Comitato Etico Area Vasta Emilia Nord) approved the study (prot. 978/2019). Given the impossibility of retrospectively retrieving the consent for all the infants included in the study, the research ethics committee waived the need for the consent.

## 5. Conclusions

Our 12-year retrospective, observational study of culture-positive sepsis episodes in a level-three Italian NICU reported microbial distribution patterns that were comparable with data from other European studies. Our antibiotic susceptibility data support the empiric antibiotic treatment in place for EOS, and most cases of LOS, without an increased mortality. Although not observed in our study, it is known that the susceptibility patterns of some pathogens, especially those responsible for LOS, are rapidly changing, with the emerging threat of MRSA and ESBL becoming more concrete. Even more so, continuous surveillance of isolates within the NICU is essential to prevent the spread of MDR pathogens. AMS programs, together with IPC measures need to be optimized to limit selection pressure and further escalation in AMR.

## Figures and Tables

**Table 1:** Period 1 and 2 overview (episodes < 34 weeks, <1500 g, EOS vs. LOS, Gram-positive vs. Gram-negative, sepsis-associated mortality).

	2011	2012	2013	2014	2015	2016	Total Period 1	2017	2018	2019	2020	2021	2022	Total Period 2	Total Period 1–2
Overall episodes	10	14	13	16	23	15	91	22	17	19	14	21	31	124	215
Episodes in infants <34 weeks, *n* (%)	3 (30%)	9 (64.3%)	5 (38.5%)	9 (56.2%)	17 (73.9%)	12 (80%)	55 (60.4%)	14 (63.6%)	14 (23.5%)	12 (63.2%)	9 (64.3%)	8 (38.1%)	18 (58%)	75 (60.5%)	130 (60.4%)
Episodes in VLBW infants, *n* (%)	3 (30%)	8 (57.1%)	4 (30.7%)	10 (62.5%)	17 (73.9%)	11 (73.3%)	53 (58.2%)	14 (63.6%)	14 (23.5%)	8 (42.1%)	8 (57.1%)	8 (38.1%)	16 (51.6%)	68 (54.8%)	121 (56.2%)
EOS, *n* (%)	3 (30%)	4 (28.6%)	5 (38.5%)	4 (25%)	2 (8.7%)	4 (26.7%)	22 (24.2%)	4 (18.2%)	2 (11.8%)	2 (10.5%)	0 (0%)	4 (19%)	5 (16.1%)	17 (13.7%)	79 (36.7%)
LOS, *n* (%)	7 (70%)	10 (71.4%)	8 (61.5%)	12 (75%)	21 (91.3%)	11 (73.3%)	69 (75.8%)	18 (81.8%)	15 (88.2%)	17 (89.5%)	14 (100%)	17 (80.1%)	26 (83,9%)	107 (86.3%)	136 (63.3%)
GRAM+, *n* (%)	9 (90%)	5 (35.7%)	6 (46.1%)	8 (50%)	13 (56.5%)	7 (46.7%)	48 (52.7%)	13 (59%)	10 (58.8%)	5 (26.3%)	5 (35.7%)	14 (66.7%)	10 (32,3%)	57 (46%)	105 (48.8%)
GRAM−, *n* (%)	1 (10%)	9 (64.3%)	7 (53.8%)	8 (50%)	10 (43.5%)	8 (53.3%)	43 (47.3%)	9 (40.1%)	7 (41.2%)	14 (73.7%)	9 (64.3%)	7 (33.3%)	21 (67.7%)	67 (54%)	110 (51.2%)
Overall patients	10	14	12	16	15	11	78	19	14	15	8	14	21	91	169
Mortality, *n* (%)	2 (20%)	2 (14.3%)	2 (16.7%)	2 (12.5%)	2 (13.3%)	3 (27.3%)	13 (16.7%)	2 (10.5%)	3 (21.4%)	1 (6.7%)	0 (0%)	0 (0%)	3 (14.4%)	9 (9.9%)	22 (13%)

**Legend:** VLBW, very low birth weight; EOS, early-onset sepsis; LOS, late-onset sepsis.

**Table 2:** Resistance patterns for Gram-positive and Gram-negative isolates in **EOS**, Periods 1 and 2.

Period 1							Period 2						
**GRAM+ Isolates (*n* = 14)**	**OXA-R**	**AMP-R**	**CTX-R**	**GEN-R**	**VAN-R**	**TEC-R**	**GRAM+ Isolates (*n* = 11)**	**OXA-R**	**AMP-R**	**CTX-R**	**GEN-R**	**VAN-R**	**TEC-R**
*S. agalactiae*, *n = 9*	-	0/9 (0%)	0/9 (0%)	-	0/9 (0%)	0/9 (0%)	*S. agalactiae*, *n = 8*	-	0/8 (0%)	0/8 (0%)	-	0/8 (0%)	0/8 (0%)
*E. faecalis*, *n = 2*	-	0/2 (0%)	-	-	0/2 (0%)	0/2 (0%)	*E. faecalis*, *n = 1*	-	0/1 (0%)	-	-	0/1 (0%)	0/1 (0%)
*L. monocytogenes*, *n = 2*	-	0/2 (0%)	-	-	-	-	*L. monocytogenes*, *n = 1*	-	0/1 (0%)	-	0/1 (0%)	-	-
*S. aureus*, *n = 1*	0/1 (0%)	-	-	1/1 (100%)	0/1 (0%)	0/1 (0%)	*S. aureus*, *n = 1*	0/1 (0%)	-	-	0/1 (0%)	0/1 (0%)	0/1 (0%)
**GRAM− isolates in EOS (*n* = 8)**	**-**	**GEN-R**	**AMK-R**	**CTX-R**	**TZP-R**	**MEM-R**	**GRAM− isolates in EOS (*n* = 6)**	**-**	**GEN-R**	**AMK-R**	**CTX-R**	**TZP-R**	**MEM-R**
*E. coli*, *n = 6* ***(ESBL n = 1/6)***	-	2/6 (33.3%)	0/6 (0%)	1/6 (16.7%)	1/6 (16.7%)	0/6 (0%)	*E. coli*, *n = 4*	-	0/4 (0%)	0/4 (0%)	0/4 (0%)	0/4 (0%)	0/4 (0%)
*K. pneumoniae*, *n = 2*	-	0/2 (0%)	0/2 (0%)	0/2 (0%)	1/2 (50%)	0/2 (0%)	/	*/*	/	/	/	/	/
*/*	/	/	/	/	/	/	*P. aeruginosa*, *n = 2*	-	0/2 (0%)	0/2 (0%)	1/2 (50%)	1/2 (50%)	0/2 (0%)

**Legend**: EOS, early-onset sepsis; OXA-R, Oxacillin-resistant; AMP-R, Ampicillin-resistant; CTX-R, cefotaxime-resistant; GEN-R, gentamicin-resistant; VAN-R, vancomycin-resistant; TEC-R, teicoplanin resistant; AMK-R, amikacin resistant; TZP-R, piperacillin-tazobactam-resistant; MEM-R, meropenem-resistant; ESBL, extended-spectrum beta lactamase.

**Table 3:** Resistance patterns for Gram-positive and Gram-negative isolates in **LOS**, Periods 1 and 2.

Period 1							Period 2						
**GRAM+ Isolates(*n* = 34)**	**OXA-R**	**AMP-R**	**CTX-R**	**GEN-R**	**VAN-R**	**TEC-R**	**GRAM+** **Isolates (*n* = 46)**	**OXA-R**	**AMP-R**	**CTX-R**	**GEN-R**	**VAN-R**	**TEC-R**
*E. faecalis*, *n* = 12	-	0/12 (0%)	-	-	0/12 (0%)	0/12 (0%)	*E. faecalis*, *n* = 19	-	0/19 (0%)	-	-	0/16 (0%)	0/16 (0%)
*S. aureus*, *n* = 13 ***(MRSA, n =*** **2/13*)***	2/8 (25%)	-	-	2/13 (15.3%)	0/13 (0%)	0/13 (0%)	*S. aureus*, *n* = 19 ***(MRSA, n =*** **4/19*)***	4/19 (21%)	-	-	4/19 (21%)	0/19 (0%)	0/19 (0%)
*S. agalactiae*, *n* = 8	-	-	-	-	0/8 (0%)	0/1 (0%)	*S. agalactiae*, *n* = 7	-	-	-	-	0/7 (0%)	0/2 (0%)
*L. monocytogenes*, *n* = 1	-	-	-	-	-	0/1 (0%)	*L. monocytogenes*, *n* = 0	/	/	/	/	/	/
*S. pneumoniae*, *n* = 0	/	/	/	/	/	/	*S. pneumoniae*, *n* = 1	-	-	0/1 (0%)	-	0/1 (0%)	-
**GRAM−** **Isolates (*n* = 35)**	**-**	**GEN-R**	**AMK-R**	**CTX-R**	**TZP-R**	**MEM-R**	**GRAM−** **Isolates (*n* = 61)**	**-**	**GEN-R**	**AMK-R**	**CTX-R**	**TZP-R**	**MEM-R**
*K. pneumoniae*, *n* = 12 ***(ESBL =*** **1/12*)***	-	1/12 (8.3%)	0/12 (0%)	1/12 (8.3%)	2/12 (16.7%)	0/12 (0%)	*K. pneumoniae*, *n* = 21 ***(ESBL n =*** **2/21*)***	-	0/21 (0%)	0/20 (0%)	2/21 (9.5%)	5/20 (25%)	0/20 (0%)
*E. coli*, *n* = 11 ***(ESBL n =*** **2/11*)***	-	1/11 (9%)	0/11 (0%)	2/11 (18.2%)	0/11 (0%)	0/11 (0%)	*E. coli*, *n* = 13 ***(ESBL n =*** **1/13*)***	-	5/13 (38.5%)	2/13 (15.4%)	1/13 (7.8%)	3/13 (23.1%)	0/13 (0%)
*P. aeruginosa*, *n* = 4	-	0/4 (0%)	0/4 (0%)	-	1/4 (25%)	0/4 (0%)	*P. aeruginosa*, *n* = 8	-	0/8 (0%)	0/8 (0%)	-	1/8 (12.5%)	0/8 (0%)
*K. oxytoca*, *n* = 3	-	0/3 (0%)	0/3 (0%)	0/3 (0%)	0/3 (0%)	0/3 (0%)	*K. oxytoca*, *n* = 5 ***(ESBL n =*** **1/5*)***	-	0/5 (0%)	0/5 (0%)	1/5 (20%)	1/5 (20%)	0/5 (0%)
*E. cloacae*, *n* = 3	-	1/3 (33.3%)	1/3 (33.3%)	0/3 (0%)	0/3 (0%)	0/3 (0%)	*E. cloacae*, *n* = 10	-	0/10 (0%)	0/10 (0%)	0/10 (0%)	1/10 (10%)	0/10 (0%)
*S. marcescens*, *n* = 1	-	0/1 (0%)	0/1 (0%)	0/1 (0%)	0/1 (0%)	0/1 (0%)	*S. marcescens*, *n* = 2	-	1/2 (50%)	0/2 (0%)	0/2 (0%)	0/2 (0%)	0/2 (0%)
*A. baumannii*, *n* = 1	-	0/1 (0%)	0/1 (0%)	0/1 (0%)	-	0/1 (0%)	*A. baumannii*, *n* = 1	-	0/1 (0%)	0/1 (0%)	0/1 (0%)	0/1 (0%)	0/1 (0%)
*C. freundii*, *n* = 0	/	/	/	/	/	/	*C. freundii*, *n* = 1	-	0/1 (0%)	0/1 (0%)	0/1 (0%)	0/1 (0%)	0/1 (0%)

**Legend:** LOS, late-onset sepsis, OXA-R, Oxacillin-resistant; AMP-R, Ampicillin-resistant; CTX-R, cefotaxime-resistant; GEN-R, gentamicin-resistant; VAN-R, vancomycin-resistant; TEC-R, teicoplanin resistant; AMK-R, amikacin resistant; TZP-R, piperacillin-tazobactam-resistant; MEM-R, meropenem-resistant; MRSA, Methicillin-resistant *S. aureus*; ESBL, extended-spectrum beta lactamase, CRE, Carbapenem-resistant *Enterobacterales.*

**Table 4:** Comparison of primary and secondary outcomes between Periods 1 and 2.

		Period 1 (2011–2016)	Period 2 (2017–2022)	*p*-Value
*Primary outcomes*	*Sepsis-associated mortality*, *n (%)*	13/78 (16.7%)	9/91 (9.9%)	0.2
	*MRSA infection rate*, *n (%)*	2/91 (2.2%)	4/124 (3.2%)	0.6
	*VRSA infection rate*, *n (%)*	0/91 (0%)	0/124 (0%)	/
	*VRE infection rate*, *n (%)*	0/91 (0%)	0/124 (0%)	/
	*ESBL infection rate*, *n (%)*	4/91 (4.3%)	4/124 (3.2%)	0.6
	*CRE infection rate*, *n (%)*	0/91 (0%)	0/124 (0%)	/
	*CRPA infection rate*, *n (%)*	0/91 (0%)	0/124 (0%)	/
	*CRAB infection rate*, *n (%)*	0/91 (0%)	0/124 (0%)	/
*Secondary outcomes*	*Bacterial sepsis episodes in patients < 34 weeks*, *n (%)*	55/91 (60.4%)	75/124 (60.5%)	0.9
	*Bacterial sepsis episodes in patients < 1500 g*, *n (%)*	53/91 (58.2%)	68/124 (54.8%)	0.6

**Legend:** MRSA, Methicillin-resistant *S. aureus*; VRSA, Vancomycin-resistant *S. aureus*, VRE, Vancomycin-resistant *Enterococci*; ESBL, extended-spectrum beta-lactamase, CRE, Carbapenem-resistant *Enterobacterales*.

## Data Availability

Rough data supporting reported results are made available upon reasonable request to the corresponding author.
